# GeneWeaver: finding consilience in heterogeneous cross-species functional genomics data

**DOI:** 10.1007/s00335-015-9575-x

**Published:** 2015-06-20

**Authors:** Jason A. Bubier, Charles A. Phillips, Michael A. Langston, Erich J. Baker, Elissa J. Chesler

**Affiliations:** The Jackson Laboratory, Bar Harbor, ME 04609 USA; Department of Electrical Engineering and Computer Science, University of Tennessee, Knoxville, TN 37996 USA; Computer Science Department, Baylor University, Waco, TX 76798 USA

## Abstract

A persistent challenge lies in the interpretation of consensus and discord from functional genomics experimentation. Harmonizing and analyzing this data will enable investigators to discover relations of many genes to many diseases, and from many phenotypes and experimental paradigms to many diseases through their genomic substrates. The GeneWeaver.org system provides a platform for cross-species integration and interrogation of heterogeneous curated and experimentally derived functional genomics data. GeneWeaver enables researchers to store, share, analyze, and compare results of their own genome-wide functional genomics experiments in an environment containing rich companion data obtained from major curated repositories, including the Mouse Genome Database and other model organism databases, along with derived data from highly specialized resources, publications, and user submissions. The data, largely consisting of gene sets and putative biological networks, are mapped onto one another through gene identifiers and homology across species. A versatile suite of interactive tools enables investigators to perform a variety of set analysis operations to find consilience among these often noisy experimental results. Fast algorithms enable real-time analysis of large queries. Specific applications include prioritizing candidate genes for quantitative trait loci, identifying biologically valid mouse models and phenotypic assays for human disease, finding the common biological substrates of related diseases, classifying experiments and the biological concepts they represent from empirical data, and applying patterns of genomic evidence to implicate novel genes in disease. These results illustrate an alternative to strict emphasis on replicability, whereby researchers classify experimental results to identify the conditions that lead to their similarity.

## Heterogeneous functional genomics data integration in GeneWeaver

There is a growing need to find consensus among large collections of diverse biological data to relate molecular mechanisms to disease and to understand the relations among diverse disease characteristics, models, and mechanisms through their underlying biology. Researchers have an ever-expanding set of technologies for the characterization and quantification of genetic variants, gene products, and other biological molecules associated with disease. This genome-wide experimentation has produced a substantial quantity of diverse data on the associations among genetic variants, model organism phenotypes, gene expression patterns, and many other disease-related endpoints. Typically, the outcomes of functional experiments consist of a set of genomic features, often genes or transcripts, but increasingly other entities such as allelic variants, methylation sites, small non-coding RNA, and an expanding variety of new biomolecular endpoints. Each of these is associated with a biological or behavioral assay, which is in turn associated with a disease-related construct. Often, the evidences for such associations vary across species and experimental paradigms. This variation can help unravel relationships among biological processes and diseases, facilitate an improved characterization of disease, identify precision animal models of human disease, and refine classification of ambiguous diseases. Ultimately, these developments will lead to refined recognition of biological mechanisms, diagnostics, and therapeutics through detection of conserved roles of genes in multiple biological contexts.

**Fig. 1:**
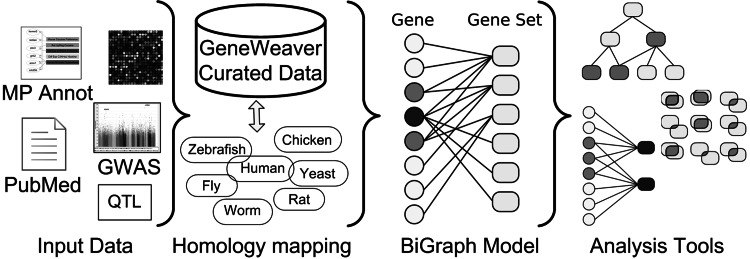
An overview of the GeneWeaver system. Diverse experimentally derived sets of gene products are curated from the literature, obtained from major public resources, or uploaded by users for shared or private use. These data are harmonized through a graph of gene identifiers and cross-species homology mappings. The harmonized gene sets are represented as a bipartite graph of genes and gene sets and analyzed using a suite of tools

**Fig. 2:**
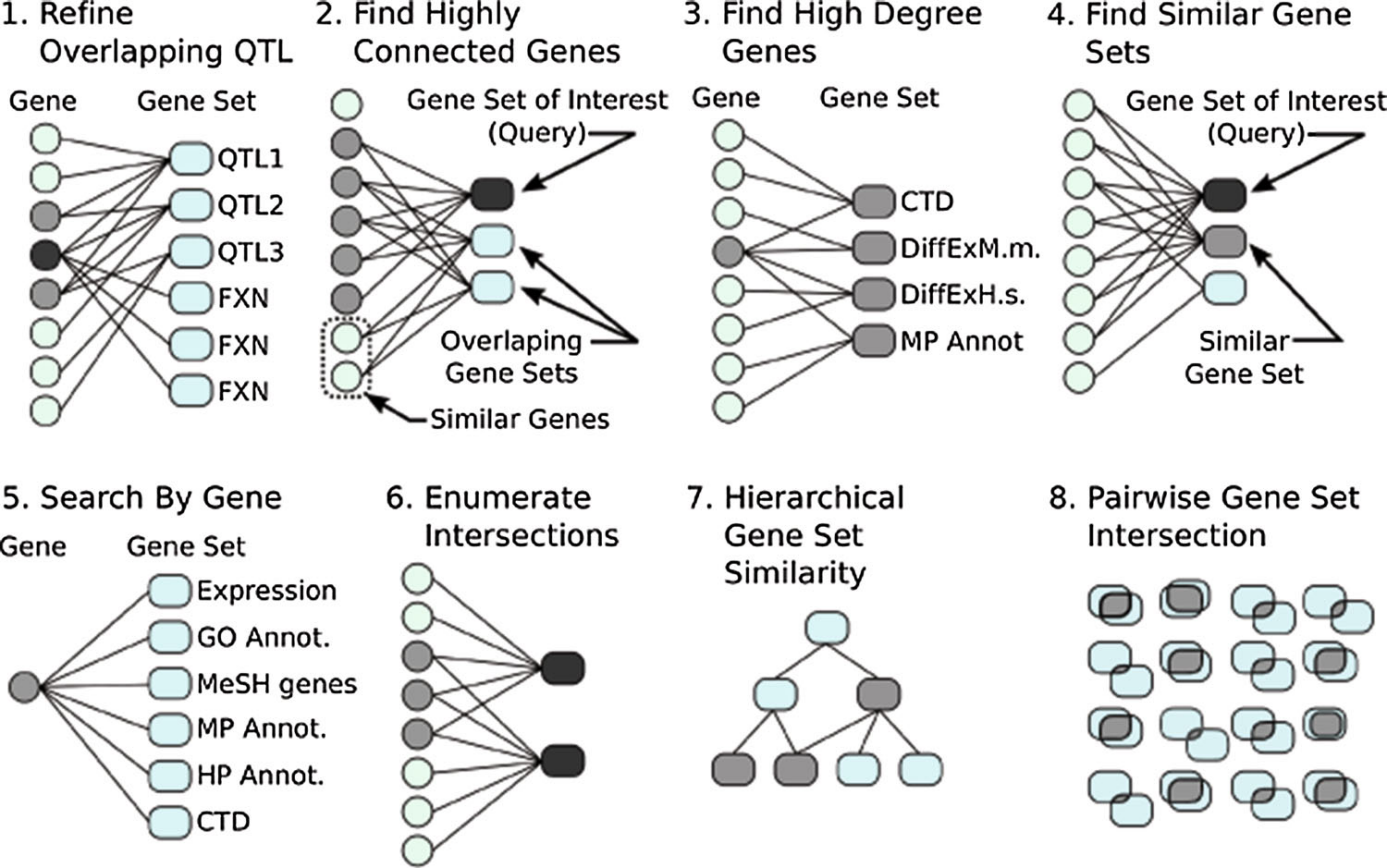
Biological applications of the GeneWeaver bipartite graph representation. A bipartite graph consists of two partite sets of vertices, with edges between but not within each partite set. A GeneWeaver collection of gene sets consists of one such partite set of vertices representing gene set identifiers and the other partite set representing the genes contained in all the gene sets. * Edges * between a gene set and genes define gene set membership. This discrete mathematical structure makes possible an efficient application of specialized graph algorithms for rapid comparisons among large numbers of gene sets. (*1*) To refine overlapping QTL and prioritize functional candidates using trait-relevant data, positional candidates for each locus are entered as gene lists (QTL1-3) and compared to genomic studies of related traits (FXNs). Genes within overlapping QTL are represented in* gray*, a functionally relevant shared candidate is indicated in* black*. (*2*) The ABBA tool is used to find similar genes based on a guilt-by-association-type transitive inference. A gene set of interest is entered into ABBA. Gene sets that overlap the input set either directly or through gene homology among the elements of the sets are retrieved (*blue oval* nodes). Genes and homologs which are highly similar to the input set based on shared connectivity are retrieved. (*3*) Highly connected (i.e., high degree) genes are found using the gene set graph tool. A group of gene sets are selected from user uploads or search results. The Gene Set graph represents the most highly connected genes with a user-defined threshold for minimum degree (number of edges) from each gene. The highest degree genes are forced to the* right* of the plot (although not shown here). (*4*) To find similar gene sets, users calculate the Jaccard similarity of each gene set in the database to a single user-selected gene set. Results are presented in a ranked table. (*5*) Entering a single gene identifier in the search box generates a list of gene sets containing the query gene or any homolog or identifier match to the gene. (*6*) To find the gene elements in the intersection of gene sets, users select a Jaccard similarity value from any table or matrix. (*7*) The Hierarchical Gene Set Similarity Graph represents successively higher order intersections in a directed acyclic graph, such that individual lists are at the leaves of the graphs and two-way, three-way…n-way intersections are represented on increasingly higher levels of the graph. Shading represents nodes that contain members of a user-selected set of ‘emphasis genes.’ (*8*) Pairwise gene set intersections are analyzed using the ‘Jaccard similarity’ or ‘Hypergeometric test’ tools. The positive matches (intersection) are compared to the set of possible matches for each pair of gene sets (Color figure online)

The GeneWeaver software system (Baker et al. 2012) contains a database and a suite of tools to enable dissemination, integration, analysis, and discovery from heterogeneous data derived through functional genomics experimentation and biological data curation (Fig. 1). There are many paths of inquiry made possible with heterogeneous functional genomics data, each aimed at discovery of convergent evidence for gene–gene, gene–disease, or disease–disease relations. As data from gene expression and genetic mapping studies accumulate, investigators have realized the potential for statistical meta-analysis to refine presumptive associations of genetic loci and disease. Recent efforts aim to discover the differences among studies in which the effects occur (Kang et al. 2014). Most collections of functional genomics data sets related to a specific biological or behavioral concept are highly heterogeneous, however, and therefore are not amenable to quantitative meta-analysis. In contrast, GeneWeaver supports this data diversity through combinatorial integration of heterogeneous data, enabling cross-species, cross-platform comparison of multiple experiments. GeneWeaver currently supports data sets from nine species (*Mus musculus, Homo sapiens, Rattus norvegicus, Danio rerio, Drosophila melanogaster, Macaca mulatta, Caenorhabditis elegans, Saccharomyces cerevisiae*, and *Gallus gallus).* Many applications are made possible through the representation of gene sets as graphs (Fig. 2). The system provides a database of curated and user-submitted sets of genes defined through a variety of processes, including differential expression experiments, QTL positional candidates, genes annotated to mutant phenotypes, global analysis of gene annotations to MEDLINE abstracts, genetic mapping, and numerous other approaches. Many gene sets are obtained from functional annotations in model organism databases, including the Mouse Genome Database. A system of users, groups, and projects allows for persistent storage or user selections, progressive reanalysis, and result sharing. Although most of these sources and GeneWeaver’s current tool suite operate on lists of genes and their association scores (*p* value, q-value, correlation coefficient, etc.), an upcoming release will support storage of gene–gene relations to provide for the direct comparison of biological networks.

Each gene set in GeneWeaver is stored with both controlled annotation, free-text description and a curation Tier designation to facilitate retrieval and filtering of relevant studies (Baker et al. 2012). Descriptive meta-content consists of a gene set name, publication information including title and abstract, gene set description which provides detail about the criteria and methods by which genes are assigned to the gene set, a short abbreviation to aid in visualization labels, and annotations to structured vocabularies (Disease Ontology, Open Biomedical Investigations, anatomy, and others) based on automated scan of the text description, and modified through manual curation. Gene sets are denoted by one of five curation tiers (Baker et al. 2012), Tier I designates data derived from curated resources, e.g., Gene Ontology annotations found in model organism databases, Tier II designates data derived from an operation performed on resource data, e.g., genetic correlations to gene expression calculated from GeneNetwork.org, Tier III are gene sets curated from literature or public data submissions that pass curatorial review, Tier IV are public data submissions that have not yet passed curatorial review, and Tier V are data sets uploaded for private use by users and groups.

The structure and contents of the GeneWeaver system have been previously described (Baker et al. 2012), as have the methods for identifier alignment (Jay 2012) and combinatorial analysis (Baker et al. 2009, 2012; Zhang et al. 2014). Tutorials for the use of the system are provided at Geneweaver.org/wiki, and the execution steps for a few applications have been described in detail (Jay and Chesler 2014). Here, we highlight some of the biological applications of the GeneWeaver system as employed by users for finding and navigating convergent evidences in diverse functional genomics data.

## Comparing experimental results using gene set intersections

GeneWeaver has several tools that facilitate comparison of studies through set–set overlap among collections of gene sets. Simple pairwise comparisons among a user-selected or user-submitted collection of gene sets can be made using the Jaccard Similarity tool. More complex intersections can be enumerated through the hierarchical similarity graph and Boolean algebra functions. Using these capabilities, Dever and colleagues retrieved and reanalyzed gene expression data from NCBI’s Gene Expression Omnibus system GEO (GSE35864) and uploaded it to GeneWeaver to identify genes differentially expressed between HIV-negative and HIV encephalitis-positive patients across multiple brain regions, including increased expression of the opioid receptor, MOR-1K (Dever et al. 2014). This indicated a potential mechanism by which morphine use may have exacerbated effects in patients with HIV encephalitis. Another example demonstrates how functional genomic analysis can enable the characterization of poorly understood drug effects, possibly discovering new applications to related diseases. GeneWeaver analysis was applied to understand the effects of Fingolimod (FTY720), currently used as a treatment for multiple sclerosis. By uploading their set of genes differentially expressed in brains of mice treated with this drug, the authors compared this set with results from other experiments in GeneWeaver, thus enabling identification of a mechanistic role for this drug in decreased histone acetylation, learning, and memory, and ultimately leading to the suggestion that this drug may be clinically useful in the extinction of aversive memories (Hait et al. 2014).

## Searching for novel disease-relevant genes with a conserved role across species

The aggregation of gene sets reveals conserved, frequently occurring but previously un-annotated genes related to biological concepts reflected by the user queries of the database or other collections of gene sets developed or curated by users around a concept of interest. Many genes on these lists are implicated in multiple studies, but have weak or non-existent evidence from deep biological investigations. For example, a cross-species collection of gene sets related to “alcoholism” revealed a highly connected gene associated with alcohol drinking, alcohol response, and alcoholism in multiple species that has no prior association to alcohol phenotypes in mice through MP annotations or alcoholism in humans via OMIM annotations, whereas known alcohol-related genes are found only in a small subset of the retrieved gene lists (Bubier and Chesler 2012). In another investigation, gene set intersection was used to combine and rank results from multiple microarray studies of alcohol-related gene expression in mouse and differential expression in human alcoholic and control brains. From this cross-species analysis, a novel role was discovered for the gene *Clic4,* which was verified in vivo as modulating alcohol behaviors in fly, worm, and mouse (Bhandari et al. 2012).

## Mapping mouse models onto human disease

A fundamental challenge in harnessing animals for the study of human disease is to understand and identify precise and relevant models. GeneWeaver aids this process through the comparison of genomic correlates of disease across species. In this approach, no assumptions of face validity are required. Rather, the underlying construct reflected in molecular correlates of the disease are directly matched to find a biologically relevant model. For example, one may start with a list of genes associated with human disease and compare the list to GeneWeaver’s database of mouse, rat, fly, yeast, worm, and other genomes to find similar gene sets. Utilizing gene2pubmed data (Maglott et al. 2011), publicly available from NCBI, and Pubmed MeSH associations, we generated publicly available GeneWeaver gene sets associated with each MeSH term. A search for similarity to the set of genes annotated to the MeSH term “alcoholism” (GS128735) resulted in the retrieval of a number of relevant sets, including GS216653, protein biomarkers of alcohol abuse (Torrente et al. 2012), and GS137124, differentially expressed genes in *Celf4* mutants (Wagnon et al. 2012). We therefore predict that this mouse model is prone to increased alcohol consumption, a previously undiscovered relationship.

## Characterizing gene function through search of GeneWeaver

The GeneWeaver database of greater than 75,000 gene sets is searchable by gene or terms found in free-text descriptions. This enables users to find gene sets containing a gene of interest and thereby identify potential functional roles of the gene across species or provide additional convergent evidence for the role of the gene in a known process. For example, in a study of the genetics of muscle and fat composition in cattle, Cesar and colleagues (Cesar et al. 2014) identified LOXL2 as a positional candidate within a QTL and used the GeneWeaver system to determine that it is also within the positional candidates set for the related phenotype, lean body mass, mapped in *Mus musculus* QTL, *Lbm10* (GS136088), originally reported by Masinde et al. (Masinde et al. 2002).

**Fig. 3:**
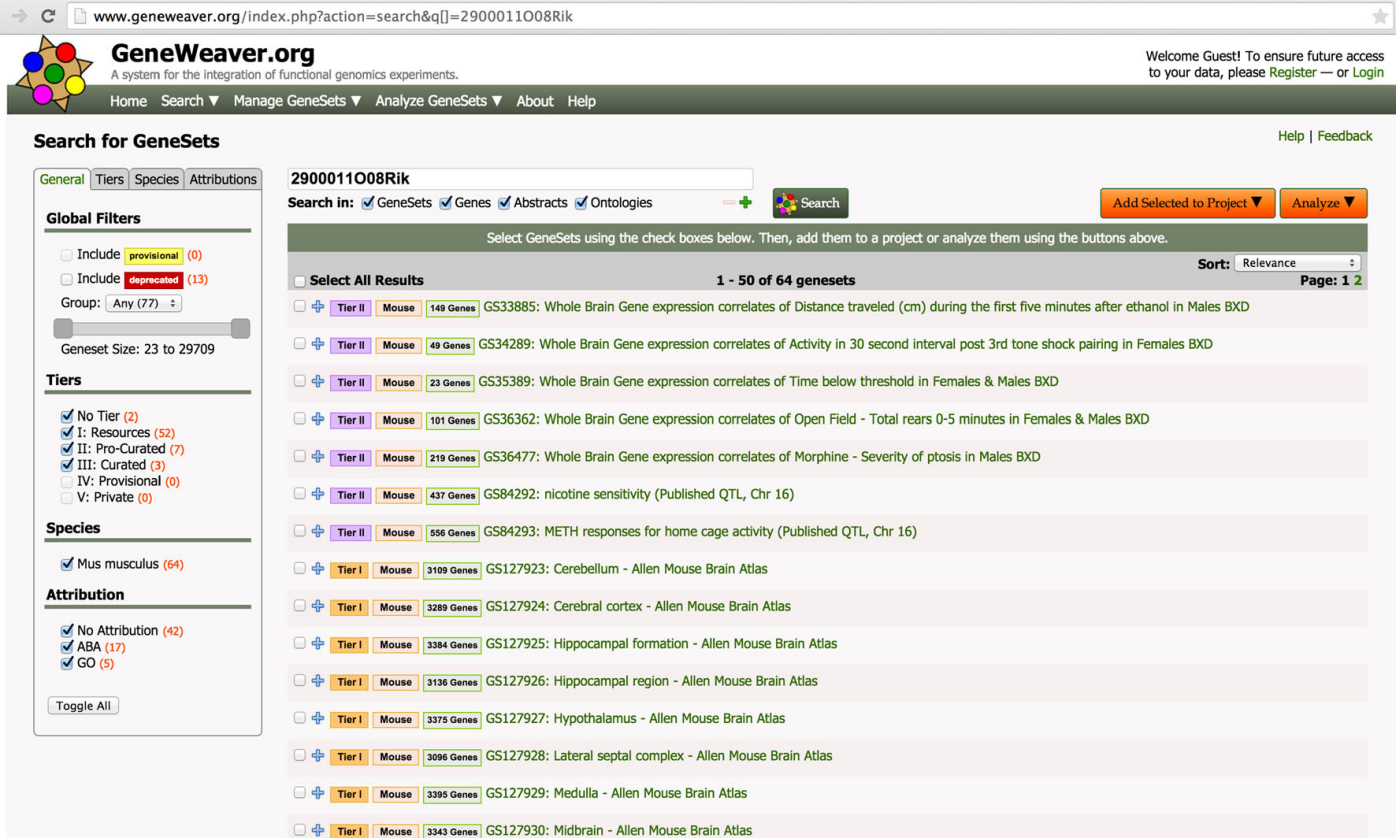
Search for a single poorly annotated transcript, *2900011O08Rik*, results in the retrieval of many gene sets that together reveal experimentally derived insights into its putative role in addiction and other related behaviors

A single gene query of the system is perhaps most compelling when unknown or poorly characterized genes are evaluated. These so-called “functionally enigmatic genes” can be interrogated to identify patterns among the gene sets in which they appear. For example, the brain ignorome (Pandey et al. 2014), represented in GeneWeaver gene sets GS218259 and GS218282, can be interrogated gene-by-gene to identify stored gene sets that may indicate putative functional roles of the query gene in biological processes. Such analysis can also reveal subtle disease-associated concepts represented in specific experimental data. A search for one such gene (*2900011O08Rik*) reveals that it is worthy of study in addiction-related behaviors (Fig. 3). It is in the QTL for multiple addiction-related traits, including nicotine sensitivity, GS84292 (Gill and Boyle 2005), methamphetamine-induced home cage activity, GS94293 (Grisel et al. 1997), acute functional tolerance to alcohol, GS135263 (Bennett et al. 2007), locomotor activity, GS136175 (Kelly et al. 2003), and nicotine-induced locomotor activity, GS136334 (Boyle and Gill 2009). It is a gene expression correlate of alcohol-induced locomotor activation (GS33885), morphine-induced Ptosis (GS35477), rearing in the open field (GS36362) in BXD recombinant inbred mice (Philip et al. 2010), and haloperidol-induced catalepsy (GS216896) from a study of selected lines (Iancu et al. 2012). It is expressed in multiple brain regions, including the striatum (GS127938), thalamus (GS127939), amygdala (GS127937), pons (GS127933), pallidum (GS127932), midbrain (GS127930), hippocampus (GS129926), cerebellum (GS127923), cerebral cortex (GS127924), and others (Lein et al. 2007). It is also a positional candidate for thalamus volume, gray matter volume, and water maze search time (GS129085) (Dong et al. 2007). The IMPC collection of deletion knock-outs (Koscielny et al. 2014) provides a readily accessible resource in which to evaluate the role of this gene in functional studies, and the information gleaned from aggregate functional genomics evidence in GeneWeaver suggests specific addiction-related phenotypes for evaluation.

In another application of gene-by-gene search, GeneWeaver was used to obtain additional functional information for a list of gene expression correlates of the duration of grooming behavior in mice. In an effort to determine which neural processes are associated with grooming, a behavior which the authors assert may be related to human anxiety disorders, Roth and colleagues (Roth et al. 2013) searched for gene sets containing each individual gene on their list of correlates that had a known human homolog. Using this approach, they were able to find 27 of the 31 correlated genes in gene sets related to mouse neural phenotypes, thereby refining a large list of correlates to those most functionally relevant. These genes were analyzed further in other bioinformatics tools resulting in discovery of a single cross-species interactome network that represents promising translational targets to study grooming behavior.

## Evaluating the role of specific genes and diseases through search of GeneWeaver

By refining the gene-centric query with a functional term, the putative role of a gene in specific traits can be evaluated. For example, in an analysis of the role of voltage-gated potassium channels in alcoholism (Padula et al. 2015), GeneWeaver was queried for ‘Kcnn1-3 AND alcohol’ plus ‘Kcnn1-3 AND drug’ resulting in retrieval of 26 gene sets from 16 different publications, including many behaviors related to alcohol, nicotine, and illicit drugs. Further analysis of these gene sets using the Gene Set Graph function enabled the identification of 8 other genes that were present in 70 % of the retrieved results and are therefore likely to function with *Kcnn3* in addiction-related phenotypes.

## Finding functionally related genes through a gene set graph walk

A “guilt-by-association” analysis of gene function is formalized in GeneWeaver’s Anchored Bicliques of Biomolecular Associations (ABBA) tool, which performs a graph walk that starts from a known gene or gene set and transitions to similar genes through shared phenotype connectivity. Currently, this tool uses an unweighted graph, but future releases are planned to make use of weighted graph walks. In its current implementation, the query consists of a gene set of interest. In the first step, a search is performed to find any gene sets that contain a user-specified minimum overlap with genes in the query list. In the second step, ABBA then provides a ranked list of genes based on their prevalence in the resulting gene sets that are not found in the initial list. The resulting genes have similar gene set connectivity with the original input set, but may not have been previously considered in relation to the biological concept represented by the input gene set. Using ABBA, Chan et al. was able to find the genes that were most often found in association with their gene of interest, *Clic4*. They identified 157 genes which were taken forward in their analysis to understand the role of the *Clic4* network in ethanol sensitivity and tolerance (Chan 2013).

Another application of the ABBA tool is in targeted literature curation based on gene similarity to existing disease-associated genes. In one such example, an ABBA search was seeded with genes annotated to autism-like phenotypes in mouse models. The most frequently occurring new genes from gene sets containing multiple input genes were used by MGI curators in a targeted literature search for relevance to autism, resulting in annotation of two additional mutant mouse alleles of *Unc5c* and *Plcb4* which were annotated to autism-related phenotypes in the MGI database (Meehan et al. 2011).

The ABBA tool can also be used to augment information found in collections of GeneWeaver gene sets. For example, a time series analysis of gene expression in the cerebellum of developing mouse *Pax6* and *Atoh1* mutant mice and their wild-type controls was deposited in GeneWeaver (Ha et al. 2012). A derived gene set formed using the Boolean tool, GS222606, contains 21 genes found in the overlap of the two mouse mutant expression studies. Analysis of this set as an input to the ABBA tool resulted in the retrieval of 483 gene sets containing at least 10 of the input genes and identification of six additional genes that are highly prevalent among the retrieved gene sets. A review of phenotypic alleles of these genes for ‘abnormal brain morphology’ in Mouse Genome Database reveals that several already have a known role in cerebellar development: *Vldlr* (Trommsdorff et al. 1999), *Ntrk2* (Minichiello et al. 1998*)*, *Vegfa* (Ferrara et al. 1996), and *Rora* (Sidman et al. 1962). Two additional genes, *Camk2b* and *Apoe,* to date have not been connected to murine cerebellar development in the literature and should be evaluated experimentally.

## Refinement of QTL positional candidates through integration of heterogeneous functional evidence

Several other studies have used GeneWeaver to refine QTL positional candidates. This has been done for alcohol-related traits in conventional F2 hybrid crosses (Chesler et al. 2012) and more complex populations including a nociception study in the Diversity Outbred (Recla et al. 2014). In one study, regional gene expression data derived from the Allen Brain Atlas were integrated with QTL positional candidates to identify plausible genetic loci that may regulate the volume of the lateral septal nucleus and associated behaviors (Talishinsky and Rosen 2012).

GeneWeaver provides straightforward integration and resolution of genetic mapping studies of related phenotypes across multiple populations through cross-species synteny and overlap of loci with distinct patterns of allelic variation across strains and across populations. This provides genetic refinement of the often large loci associated with complex traits. Although the concept of multiple-cross QTL comparison is not new (Hitzemann et al. 2002, 2008 ; Li et al. 2011), assembling the resources, aligning genetic loci, and refining sets of variants is cumbersome and requires significant effort or advance planning. By integrating the Mouse Genome Database, Rat Genome Database, and other curated repositories of genetic loci into GeneWeaver, rapid retrieval and cross-trait, cross-population, and cross-species comparison has been made possible. For example, Mulholland and colleagues were able to compare overlapping mouse QTLs from multiple crosses to identify putative variants in *Kcnq2* and their potential role in independently mapped alcohol-related phenotypes (McGuier et al., in press).

Comparison of strain distributions across multiple mouse crosses, particularly with some overlap in founders, can even enable refinement to the level of single-nucleotide polymorphisms. For example, a recent study (Bubier et al. 2014) demonstrates the use of GeneWeaver in combination with additional precision genetic resources and genomic databases to identify the precise single-nucleotide polymorphism conferring variation in epigenetic regulation of gene expression as a mechanism for genetic variation in two related biobehavioral processes, alcohol preference and withdrawal. In the first stage of this work, sets of genes related to each function were retrieved and combined to find highly connected preference- and withdrawal-associated genes. In the second stage, these were intersected to reveal a single candidate, *Ap3m2*, which was then subject to specific database interrogation and other tests of genetic and functional validity, ultimately enabling us to identify a differentially methylated SNP underlying the observed QTLs. Notably, this example illustrates how very large, unresolved legacy results can be integrated and refined to reveal molecular mechanisms for complex traits. Many other sets of highly overlapping loci for related traits can be found in GeneWeaver, facilitating application of this approach to a wide variety of diseases.

## Sharing and augmentation of experimentally derived functional genomics data

The GeneWeaver system allows users to upload and share genome-wide outcomes of experimentation, in contrast to conventional archives for storage of raw experimental data. Sharing and archiving experimental results allows users to readily examine relationships among genome-wide outcomes from very large collections of studies without the need to retrieve and reanalyze each individual study. For example, in a study of the molecular mechanisms of hybrid sterility, Bhattacharyya and colleagues (Bhattacharyya et al. 2013) submitted microarray data to NCBI’s Gene Expression Omnibus system (GSE41707) to make raw data available. They also self-archived four enriched gene sets (GeneWeaver GS213073-GS213077) specifically reflecting expression differences among sterile hybrids and fertile controls. Each set represents the comparison of cross founders and F1 progeny.

By placing these data in GeneWeaver, additional analyses are readily possible. For example, a hierarchical intersection analysis reveals 471 genes found on all four lists. This new list was used to query the database for similar gene sets based on Jaccard similarity to all database contents. This search revealed overlap of the differentially expressed genes with positional candidates within a QTL for testes weight, GS136884 (Zidek et al. 1998), and GO annotations to pheromone receptor activity and response (GS180456, GS1192092). Eleven of these gene sets were selected and placed in a GeneWeaver project. The original intersection sets were specified as ‘emphasis genes’ to highlight them in results output. A hierarchical similarity analysis and gene set graph analysis reveals that *Vmn1r199* and other pheromone receptors are highly connected genes, suggesting that one candidate mechanism of infertility is disruption in pheromone signaling and its potential effects on reproductive behavior. An additional overlapping subnetwork includes two reproductive QTLs mapping to the *M.musculus* Chromosome X, containing positional candidates *Spin2a*, *Diap2*, and *Taf7* *l*. Of these genes, *Diap2* has been associated with female sterility (Bione et al. 1998) and *Taf7l* has been associated with male sterility (Akinloye et al. 2007; Stouffs et al. 2006). Because these QTLs were mapped in multiple crosses, it may be possible to identify candidate genetic variants in the Sanger Mouse Genomes sequence resource (Keane et al. 2011) to further refine the causal variants in these loci. Furthermore, the identification of overlapping and functionally relevant gene sets provides insight into additional biological endpoints, beyond sterility, that may be influenced by such variants.

## Data-driven disease classification

The aggregate analysis of heterogeneous data from diverse, genome-wide genomic associates of complex disease provides a means of data-driven classification of disease based on an overlapping biological substrate. To demonstrate the application of this strategy on which we have previously elaborated (Chesler and Logan 2012), we used GeneWeaver’s hierarchical similarity graph to partition gene annotation sets for psychiatric disorders. MEDLINE abstracts containing gene symbols and their annotations to MeSH terms were used to construct sets of genes associated with each term in the Psychiatric disorder MeSH sub-tree. Analysis in the hierarchical similarity graph tool revealed genes related to a constellation of psychiatric disorders. The tool subdivided the disorder based on 18 genes associated with ‘Anxiety’ but not ‘Depression with alcohol use’ and two associated with ‘Depression with alcohol use’ and not ‘Anxiety.’ Evaluation of *Oxtr* and *Abcb1* genes in anxiety and alcohol assays, along with representatives of the 18 anxiety genes not associated with depression and alcohol dependence, will test the validity of this classification. One caveat to note is that in an effort to reduce artifacts, genes were only associated to a particular MeSH term if they occurred in a minimum of two publications associated to that MeSH term. As a result, GeneWeaver may find partitions based on missing or weak connectivity, i.e., absence of evidence, not evidence of absence. The precision and accuracy of this strategy should be greatly improved over time with the inclusion of larger collections of data.

## Interpreting and comparing gene co-expression networks

In most of the examples we have provided, sets of genes are compared and contrasted. This gene set integration provides a versatile approach to dealing with most functional genomics result types, but increasingly network analysis methods provide even greater precision. Many of the technologies used to generate lists of biologically important genes are error prone. The more stringent criteria provided by gene–gene correlation or interaction provide greater evidence of a common role of genes in a process. Gene co-expression network analysis is widely employed to find modules of genes that behave in concert in relation to biological manipulations or across segregating populations. Although these are often represented as lists or sets of module members, they implicitly reflect high interconnectivity among the members. We have previously shown that dense co-expression sub-networks have high biological relevance, and the interpretation of individual subgraphs can reveal functional roles of these modules.

Gene co-expression modules from a study of haloperidol-induced catalepsy in short-term selected mouse lines (Iancu et al. 2012) were entered into GeneWeaver. The functional roles of these modules can be characterized by a search for similar gene sets. For example, one co-expression cluster, GS216879, was compared to the rest of the database. The five most similar gene sets were GS35275, striatal gene expression correlates of acoustic startle (Philip et al. 2010), GS122992, nitrosobenzylmethylamine-interacting genes from the Comparative Toxicogenomics Database (Davis et al. 2015), GS33995, correlates of blood alcohol concentration in BXD mice (Philip et al. 2010), and GS1778, differential expression in mice with high and low acute functional tolerance to alcohol (Tabakoff et al. 2003). Genes common to four of these five sets, *Timm8A* and *Gng5,* are revealed using GeneWeaver’s gene set graph tool. Other alcohol-related co-expression network studies have been placed in GeneWeaver, including co-expression of genes in BXD Recombinant inbred strains with predisposition to alcohol consumption (Vanderlinden et al. 2013).

We have recently developed a strategy to extrapolate GeneWeaver’s gene set integration tools to the comparison of network edge relations across collections of graphs (Baker et al. 2014). This will ultimately enable a direct comparison of biological networks not just through the sharing of molecular members, but also through the consistency of interactions among those molecules. Network similarity graphs will permit researchers to evaluate the presence and conservation of co-expression, molecular pathways, and molecular interaction networks across biological perturbations, genetic populations, and other heterogeneous contexts.

## Summary: finding consilience among heterogeneous experiments

Phenomenal curated biological data resources provide a rich context in which to explore functional genomics experiments. By placing the data from multiple curated databases into a single analytic framework and populating it with empirical experimental outcomes from genome-wide experimentation, data can be efficiently combined and navigated to find consilience among heterogeneous experiments. Many paths to inquiry are enabled by the system, either through the extension of a set of results or the refinement from a collection of studies. By minimizing data integration hurdles and providing a suite of set operations, users can combine and compare gene sets across experiments and species to find convergence of evidence across experiment types, model systems, organisms, and diseases.

As the highlighted examples demonstrate, the insights gained from this exploration may either be expansive, enabling users to extend existing findings by effectively navigating overlapping experimental results, or refining, reducing a wealth of genes from a single experimental result to a subset of highly supported genes across experiments. In both cases, the availability of deep and broad evidence from many sources frees the biological question from a single, restrictive context. It also provides independent, orthogonal approaches to the research question, free from the biases inherent in single measurements of a biological or behavioral concept, specific characteristics of populations including linkage disequilibrium, distributions of variants, and the presence of particular gene products in various species.

There has been a recent concern about the ability to replicate and reproduce findings in model organism studies (Seok et al. 2013; Vasilevsky et al. 2013). Reproducing experiments and replicating their results is remarkably challenging in the face of variation and limited documentation of experimental systems including model organisms, test procedures and parameters, and testing and housing environments. Resources like the Mouse Phenome Database (Grubb et al. 2014) and the International Mouse Phenotyping Consortium’s mousephenotypes.org (Koscielny et al. 2014) which follows ARRIVE guidelines (Kilkenny et al. 2010) for reporting experimental methods, provide deep insight into how data are collected in hopes that investigators will have an improved ability to reproduce each other’s experiments. Although replication of experiments in a logically consistent and falsifiable manner improves confidence in results and has been a backbone of the scientific method, the emphasis on strict replication is questionable, as the replication may be perfect; yet the generalizability of the experimental result to the research question at hand may be quite limited. This is particularly the case when exact replication of experimental conditions is nearly impossible to obtain, as in the case of laboratory environmental variation. If the objective is to test a hypothesis concerning relations among treatment conditions, environmental perturbations, and organismal variables including sex and genetic variation in a manner that translates down the hall, across the world, or to another species, one might rather seek convergence of evidence across experiments.

Seeking ‘consilience’—a common result regardless of how the question is asked or how the phenotype is assayed—may be more valuable than simply striving to replicate a system containing many unknown and poorly understood environmental and experimental variables. The main challenge is in the integration and interrogation of diverse data. Functional genomics experimentation has been described as purely inductive or ‘hypothesis generating.’ But these techniques are not merely inductive; they provide a quantitation of the state of biological systems in many contexts. Classification of the conditions under which particular results are observed provides insight into the relations among those conditions, and these relations can be subject to quantitative evaluation. GeneWeaver was designed to support the search for consilience among genetic and genomic studies of disease through the convergence of evidence in genome-wide functional genomics experiments. It provides analytic approaches to aggregate, integrate, and classify many heterogeneous experimental findings, so that we may interpret, rather than merely reject, variation in results obtained across independent studies. To quote William Whewell, “the evidence in favour of our induction is of a much higher and more forcible character when it enables us to explain and determine cases of a kind different from those which were contemplated in the formation of our hypothesis…No accident could give rise to such an extraordinary coincidence” (Whewell 1847).
